# Chromosomal rearrangement involving 11q23 locus in chronic myelogenous leukemia: a rare phenomenon frequently associated with disease progression and poor prognosis

**DOI:** 10.1186/s13045-015-0128-2

**Published:** 2015-04-08

**Authors:** Wei Wang, Guilin Tang, Jorge E Cortes, Hui Liu, Di Ai, C Cameron Yin, Shaoying Li, Joseph D Khoury, Carlos Bueso-Ramos, L Jeffrey Medeiros, Shimin Hu

**Affiliations:** Department of Hematopathology, The University of Texas MD Anderson Cancer Center, Houston, TX 77030 USA; Department of Leukemia, The University of Texas MD Anderson Cancer Center, Houston, TX 77030 USA

**Keywords:** Chronic myelogenous leukemia, *BCR*-*ABL1*, Blast phase, Clonal evolution, 11q23, *MLL*

## Abstract

**Background:**

Progression of chronic myelogenous leukemia (CML) is frequently accompanied by cytogenetic evolution, commonly unbalanced chromosomal changes, such as an extra copy of Philadelphia chromosome (Ph), +8, and i(17)(q10). Balanced chromosomal translocations typically found in *de novo* acute myeloid leukemia occur occasionally in CML, such as inv(3)/t(3;3), t(8;21), t(15;17), and inv(16). Translocations involving the 11q23, a relatively common genetic abnormality in acute leukemia, have been seldom reported in CML. In this study, we explored the prevalence and prognostic role of 11q23 in CML.

**Methods:**

We searched our pathology archives for CML cases diagnosed in our institution from 1998 to present. Cases with 11q23 rearrangements were retrieved. The corresponding clinicopathological data were reviewed.

**Results:**

A total of 2,012 cases of CML with available karyotypes were identified. Ten (0.5%) CML cases had 11q23 rearrangement in Ph-positive cells, including 4 cases of t(9;11), 2 cases of t(11;19), and 1 case each of t(2;11), t(4;11), t(6;11), and t(4;9;11). Eight cases (80%) had other concurrent chromosomal abnormalities. There were 6 men and 4 women with a median age of 50 years (range, 21–70 years) at time of initial diagnosis of CML. 11q23 rearrangement occurred after a median period of 12.5 months (range, 0–172 months): 1 patient in chronic phase, 2 in accelerated phase, and 7 in blast phase. Eight of ten patients died after a median follow-up of 16.5 months (range, 8–186 months) following the initial diagnosis of CML, and a median of 6.7 months (range, 0.8–16.6 months) after the emergence of 11q23 rearrangement. The remaining two patients had complete remission at the last follow-up, 50.2 and 6.9 months, respectively. In addition, we also identified a case with 11q23/t(11;17) in Ph-negative cells in a patient with a history of CML. *MLL* involvement was tested by fluorescence *in situ* hybridization in 10 cases, and 7 cases (70%) were positive.

**Conclusions:**

In summary, chromosomal rearrangements involving 11q23 are rare in CML, frequently occurring in blast phase, and are often associated with other cytogenetic abnormalities. These patients had a low response rate to tyrosine kinase inhibitors and a poor prognosis.

## Background

*BCR*-*ABL1* derived from t(9;22)(q34;q11), its variant translocations, or cryptic fusions is the sole chromosomal abnormality in about 80–90% of chronic mye-logenous leukemia (CML) diagnosed in chronic phase (CP). *BCR*-*ABL1* signaling is believed to be the driving force in CML pathogenesis, leading to disease progression and secondary genetic changes. Continuous *BCR*-*ABL1* activity induces DNA damage and inhibits DNA repair, leading to genetic instability and clonal evolution [1,2]. Clonal evolution, manifested by cytogenetics and mutational changes, occurs in 5–10% of patients diagnosed in chronic phase, approximately 30% of patients in accelerated phase (AP), and 50–80% of patients in blast phase (BP) [2,3]. A recent study demonstrated that the pattern of cytogenetic changes during clonal evolution remains similar in CML patients treated with or without tyrosine kinase inhibitors (TKIs), supporting the idea of genetic instability induced by *BCR*-*ABL1* as the mechanism of clonal evolution[4]. The most common chromosomal aberrations during clonal evolution are unbalanced chromosomal changes, including + Ph, +8, i(17)(q10), and +19 [4], which are the so-called “major route” abnormalities. Reciprocal translocations are much less common and represent “minor route” abnormalities. Several rearrangements typically occurring in *de novo* acute myeloid leukemia (AML) and conferring prognostic value, such as inv(3)(q21q26)/t(3;3)(q21;q26), t(8;21)(q22,q22), t(15;17)(q22;q21), and inv(16)(p13q22), have been infrequently observed as secondary cytogenetic changes during clonal evolution of CML. Reciprocal rearrangements involving 11q23 are extremely rare in CML and until now only about ten cases in the form of single case reports are available in the literature [5-14]. The clinicopathological characteristics and prognostic value of 11q23 translocations in CML have not been studied systematically.

Rearrangements involving 11q23/*MLL* locus are frequently encountered in acute leukemia. About 70%–80% of infant acute leukemia has 11q23/*MLL* abnormalities, associated with a distinct genetic profile and poor prognosis [15,16]. In adults, about 3%–4% of *de novo* AML carries 11q23 translocations [17]. Rearrangements involving 11q23/*MLL* are also observed in therapy-related AML, especially after topoisomerase inhibitor treatment. The translocation partners for 11q23 are diverse, and over 70 fusion partners have been characterized at the molecular level to date [18]. The most common translocations involving 11q23/*MLL* include t(9;11)(p22;q23), t(4;11)(q21;q23), and t(11;19)(q23;p13) [18,19]. Although the mechanisms of MLL-induced leukemogenesis seem diverse and are not fully understood, most *MLL* gene rearrangements juxtapose the amino terminal portion of MLL with the carboxyl terminal portion of its partners, resulting in chimeric oncoproteins [20]. Gene-expression profiling studies demonstrated that MLL fusion proteins induce leukemic transformation, at least in part, through activation of homeobox genes, such as *HOXA5* and *HOXA9* [21]. A recent study demonstrated that *MLL*-*AF6* fusion oncogene derived from t(6;11)(q27;q23) can induce aberrant activation of RAS and its downstream targets [22].

In this study, we aimed to explore the incidence, pathology, and clinical features of CML cases with 11q23 rearrangement.

## Results

### Incidence and clinical characteristics

A total of 2,012 cases of CML with karyotypes were identified in our pathology database and from this group, 10 cases (0.5%) carried translocations involving the 11q23 locus in Ph-positive cells. There were six men and four women with a median age of 50 years (range, 21–70 years) at the time of initial diagnosis of CML. All patients received therapies following CML diagnosis. Depending on the time of CML diagnosed, different regimens were given; historically with IFNa, and more recently with TKIs, including first-generation TKI imatinib and second-generation TKIs dasatinib and nilotinib (Table 1). In contrast to cases 1–10, case 11 was a 48-year-old male with the emergence of 11q23 translocation in Ph-negative cells.

**Table 1:** **Clinicopathological features of CML with 11q23 translocation**

**#**	**Sex/age** ^**a**^	**Treatment** ^**b**^	**Interval** ^**c**^	**11q23 phase** ^**d**^	**TKI** ^**e**^	**Treatment response**		**F/U from CML** ^**f**^	**F/U from 11q23** ^**g**^	**Status at last F/U**
						**CCyR**	**MMR**			
1	F/64	Imatinib	17	CP (1% blasts)	Yes	Yes	Yes	68	50.2	Alive
2	M/43	IFNa, Imatinib, Dasatinib	172	AP (12% blasts)	Yes	No	No	186	13.9	Dead
3	M/67	IFNa, Hydroxyurea	17	AP (12% blasts)	Yes	No	N/A	33	16.6	Dead
4	F/50	IFNa	7	BP	Yes	No	No	8	0.9	Dead
5	M/70	None	0	BP	Yes	Yes	Yes	7	6.9	Alive
6	M/31	None	0	BP	Yes	NK	NK	8	8	Dead
7	F/21	Hydroxyurea, Imatinib	12	BP	Yes	No	No	21	9.5	Dead
8	M/47	Hydroxyurea, Imatinib	13	BP	Yes	No	No	16	2.4	Dead
9	M/59	Hydroxyurea, IFNa, Imatinib	13	BP	No			14	0.8	Dead
10	F/50	Hydroxyurea, IFNa	12	BP	No			17	5.3	Dead
11	M/48	Nilotinib, Imatinib, Dasatinib	32	BP	No			36	4.1	Dead

*CCyR* complete cytogenetic response, *MMR* major molecular response, *N*/*A* not available, *NK* not known as the patient had concurrent chemotherapy with daunorubincin and ara-C.

^a^Age at initial diagnosis of CML.

^b^Initial treatments before 11q23 emergence.

^c^Interval (month) between CML diagnosis and 11q23 appearance.

^d^CML phase in which 11q23 developed.

^e^TKI treatment after 11q23 clonal evolution.

^f^Time (month) from CML diagnosis to the last follow-up.

^g^Time (month) from 11q23 emergence to the last follow-up.

### Emergence of 11q23 rearrangement in Ph-positive cells

Rearrangement involving 11q23 developed after a median period of 12.5 months (range, 0–172 months) following the initial diagnosis of CML (Table 1). It was detected in one patient (case 1) in CP, in two patients (cases 2 and 3) in AP, accompanied with other AP features including increased blast counts and thrombocytopenia; both were transformed to BP during targeted therapy with TKIs, after 1.6 and 12 months, respectively. The remaining eight patients (cases 4–10) had 11q23 rearrangement in BP; two patients (cases 5 and 6) presented as BP with 11q23 rearrangements at the initial diagnosis of CML. Both cases showed small hypolobated “dwarf” megakaryocytes. In addition, case 5 presented with basophilia (8%) and eosinophilia (4%). These features support the diagnosis of BP of CML instead of *de novo* AML with t(9;22). The other six cases developed 11q23 rearrangements during the course of CML treatment.

### Emergence of 11q23 rearrangement in Ph-negative cells

In case 11, the 11q23 translocation developed in Ph-negative cells. The patient was initially diagnosed with CML in CP, with t(9;22)(q34;q11) as a sole abnormality. He was treated with imatinib, nilotinib, and dasatinib. The patient showed a partial response to the treatment. Thirty-two months after the original CML diagnosis, the patient developed AML with the following cytogenetics: 46,XY,t(11;17)(q23;q25) [20]. No Philadelphia chromosome was identified, and fluorescence *in situ* hybridization (FISH) for *BCR*-*ABL1* was negative.

### Emergence of 11q23 rearrangement in a CML case that initially presented as myeloid sarcoma

In case 4, the patient initially presented with a breast mass that was diagnosed as myeloid sarcoma with t(9;22). The status of peripheral blood and bone marrow at that time was unknown. The patient was treated with IFNa. Three months later, bone marrow showed CML in AP, and the patient was treated with idarubicin and ara-C with a brief remission. Another 4 months later, the patient showed massive and diffuse lymphadenopathy with hepatosplenomegaly. A lymph node biopsy showed myeloid sarcoma. Bone marrow biopsy showed acute leukemia with a mixed myeloid/T phenotype (Table 2). The conventional cytogenetics of this bone marrow showed t(2;11)(q32;q23) in Ph-positive cells (Table 3).

**Table 2:** **Immunophenotype of blasts in CML with 11q23 translocations**

**#**	**Lineage**	**Blast**	**Immunophenotype**
2	Myeloid	60%	+: CD7, CD19 partial, CD13, CD33, CD34, CD38, HLA-DR, MPO
			−: CD2, CD3, CD5, CD10, CD14, CD15, CD20, CD41, CD56, CD64, CD117, TdT
3	Myeloid	58%	+: CD13, CD33, CD38, CD64, HLA-DR, MPO (cytochemistry)
			−: CD2, CD3, CD7, CD10, CD19, CD20, CD34, CD117,
4	Myeloid/T	60%	+: CD3(c), CD5, CD7, CD13, CD33, CD34, CD43, CD117, TdT, MPO
			−: CD10, CD19, CD20
5	Myeloid	64%	+: CD4 partial, CD7 partial, CD13, CD15 subset, CD33, CD38, CD64, CD123, HLA-DR, MPO
			−: CD2, CD3, CD5, CD10, CD14, CD19, CD22, CD34, CD36, CD41, CD56, CD117, TdT
6	Myeloid	80%	+: CD11c, CD13, CD33, CD34 weak, CD38, CD43, CD64, CD117, HLA-DR, MPO
			−: CD3, CD19
7	Myeloid	47% (PB)	+: CD13, CD33, CD34 partial, CD38, CD64, CD117, HLA-DR, MPO
			−: CD2, CD3, CD5, CD7, CD10, CD14, CD15, CD19, CD20, CD41, TdT
8	Myeloid	98%	+: CD13, CD33, CD34 partial, CD38, CD117, HLA-DR, MPO (3% by cytochemistry)
			−: CD2, CD5, CD7, CD10, CD14, CD19, CD20, CD41, CD64, TdT
9	Myeloid	81%	+: CD7, CD13, CD33, CD34, CD38, CD64, CD117, MPO, TdT
			−: CD2, CD3, CD10, CD19, CD20, HLA-DR
10	Myeloid	50%	+: CD10, CD13, CD22 (c), CD33, CD34, CD38, HLA-DR, TdT partial
			−: CD2, CD3, CD19, CD41, CD64, CD117, MPO
11	Myeloid	36%	+: CD4, CD13, CD15, CD33, CD34 partial, CD38, CD64, CD117, CD123, MPO
			−: CD2, CD3, CD5, CD7, CD14, CD19, CD22, CD36, CD56, HLA-DR, TdT

**Table 3:** **Karyotypes and MLL FISH in CML with 11q23 translocations**

**Case #**	**Karyotypes**	**FISH**
1	46,XX,t(4;11)(q21;q23),t(9;22)(q34;q11.2) [13] /46,XX [7]	−
2	46,XY,add(2)(p21),inv(3)(q21q26.2),der(6)t(6;11)(p11.2;q23)t(6;9)(q25;q22)t(9;22)(q34;q11.2),der(9)t(6;9),der(11)t(6;11),der(22)t(9;22) [20]	−
3	45,X,-Y,t(9;22)(q34;q11),t(11;19)(q23;p13.1) [20]	+
4	45,XX,t(2;11)(q32;q23),del(4)(p14),del(6)(q14),t(9;22)(q34;q11),-13,add(16)(q24),del(18)(q21) [20]	−
5	46,XY,t(9;22)(q34;q11.2) [16] /46,sl,t(9;11)(p22;q23),i(17)(q10) [16]/47,sl,t(9;11)(p22;q23),+17 [2]	+
6	46,XY,t(9:22)(q34;q11.2) [9] /46,XY,der(9)t(9;18)(p22;q11.2),t(9;22)(q34;q11.2),der(11)t(9;11)(p22;q23),i(17)(q10),der(18)t(11;18)(q23;q11.2),der(22)t(9;22) [11]	N/A
7	46,XX,t(9;22)(q34;q11.2) [7] /46,XX,der(4)t(4;9;11)(p12;p22;q23),der(9)t(4;9;11)t(9;22)(q34;q11.2),der(11)t(4;9;11),der(22)t(9;22) [13]	+
8	50,XY,t(9;11)(p22;q23),t(9;22)(q34;q11.2),+13,+17,+22,+der(22)t(9;22) [20]	+
9	45,X,-Y,t(9;11)(p21;q23),t(9;22)(q34;q11.2) [8] /51,idem,+X,+6,+8,+21,+22,+der(22)t(9;22) [12]	+
10	46,XX,t(9;22)(q34;q11),t(11;19)(q23;p13) [15] /46,XX [4]	+
11	46,XY,t(11;17)(q23;q25) [20]	+

### Morphology and immunophenotype

**Figure 1:**
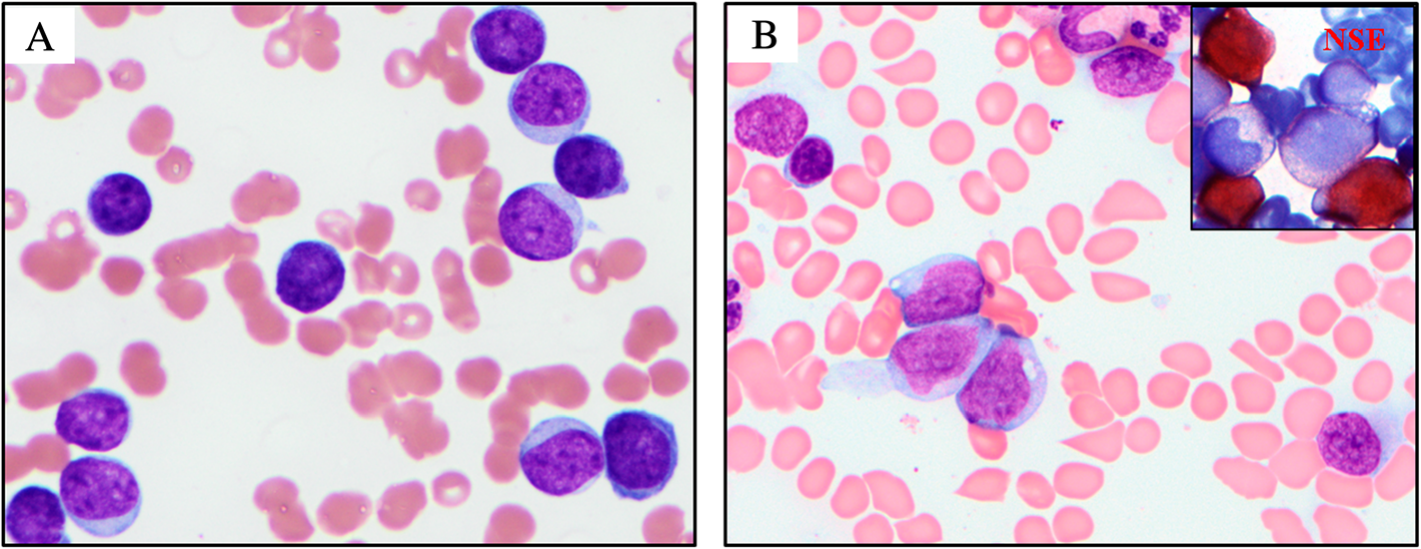
**Blast morphology in CML with 11q23,** **blast phase. (A)** A case of acute leukemia without maturation (case 8). **(B)** A case of acute leukemia with monocytic differentiation (case 7) (NSE: non-specific esterase).

Of ten cases with blast transformation in this study (cases 2–11), nine showed myeloid, and one (case 4) showed a mixed lineage (myeloid/T) immunophenotype (Table 2). Several cases expressed lymphoid markers: case 10 expressed CD10 and CD22 while case 2 had partial CD19. The blast morphology was heterogeneous and some cases have monocytic differentiation (Figure 1 and Table 2). CD64, a monocytic marker, was expressed in six cases.

### Cytogenetic data

The translocation partners for 11q23 were diverse and included four cases of t(9;11), two cases of t(11;19), and one case each of t(2;11), t(4;11), t(6;11), t(11;17), and t(4;9;11) (Table 3). In a case of t(9;11) (case 6), t(11;18)(q23;q11.2) and t(9;18)(p22;q11.2) were present, which indicates a three-way translocation (Table 3). Besides 11q23, 8 of 11 cases had additional cytogenetic abnormalities, including − Y, i(17)(q10), inv(3)(q21q26.2), etc. FISH studies to evaluate *MLL* gene status were performed in ten cases. Seven cases showed *MLL* rearrangement, whereas three cases had an intact *MLL* gene using a commercially available breakapart probe.

### Treatment and prognosis after emergence of 11q23

Eight patients (case 1–8) received TKI therapy, and three of them (cases 5–7) also received allogeneic stem cell transplant following TKI therapy. The list of TKIs treatment was case 1 with nilotinib, case 2 with nilotinib and bosutinib, case 3 with imatinib, case 4 with imatinib, case 5 with dasatinib and nilotinib, case 6 with dasatinib, case 7 with nilotinib, and case 8 with dasatinib. The remaining patients (case 9–11) received conventional chemotherapy targeting to acute leukemia.

In patients with 11q23 rearrangements in Ph-positive cells, two patients achieved complete cytogenetic response and major molecular response after TKI treatment; one patient (case 1) had 11q23 as the sole secondary abnormality and the other patient (case 5) had 11q23 at initial CML diagnosis. These two patients were alive at the last follow-up, 50.2 and 6.9 months after 11q23 emergence. The rest of the patients (8/10) died at the last follow-up, at a median of 16.5 months (range, 8–186 months) following the initial diagnosis of CML and a median of 6.7 months (range, 0.8–16.6 months) following emergence of the 11q23 translocation. The patient with 11q23 rearrangement in Ph-negative cells (case 11) died 4.1 months following emergence of 11q23 translocation.

Of the three patients who received allogeneic stem cell transplant, one patient (case 5) achieved complete remission after TKI treatment, followed by stem cell transplant. The other two patients (cases 6 and 7) had no response to TKI treatment. Transplant was performed after induction chemotherapy. Both patients had relapsed disease shortly after transplant and died at the last follow-up.

## Discussion

Although frequently encountered in AML and acute lymphoid leukemia (ALL), 11q23 rearrangement occurs very rarely in CML, in less than 1% of cases in this study by conventional karyotype. The translocation partners for 11q23 are diverse with t(9;11) (p22;q23) being the most common. Most cases developed 11q23 translocation in BP, and were often associated with other chromosomal changes. Patients had a low response rate to TKI treatment, and the overall survival was poor.

Clonal evolution with additional chromosomal abnormalities besides t(9;22) is considered as a feature of disease progression in CML. The predominant emergence of 11q23 in BP (8/11) suggests that 11q23/*MLL* may play a direct role in CML blast transformation. In AML and ALL, the role of *MLL* in leukemic transformation has been extensively studied. Although the fusion partners for *MLL* are diverse with more than 70 fusion partners described, most *MLL* rearrangements join the N-terminal region of MLL with C-terminal portion of its partners, forming chimeric oncoproteins [21]. Although the mechanisms of MLL-induced leukemia are complex and not fully understood, the chimeric proteins have been shown to induce HOX expression, which can block hematopoietic differentiation and induce leukemia [20]. Whether MLL plays a similar role in CML blastic transformation needs further confirmation.

The prognostic significance of clonal evolution in CML is variable and depends on many factors, including the particular type of cytogenetic changes, the time and phase of emergence of clonal evolution, other concurrent chromosomal abnormalities, the presence or absence of other AP features, and treatment regimens. In the era of pre-TKI therapy, INFa induced a complete suppression of clonal evolution in 46% of patients with only Ph chromosome and/or diploid karyotype present after resolution of clonal evolution. About 7% of patients with clonal evolution achieved a complete cytogenetic remission on INFa therapy, and the absence of other AP features was associated with a better response [23]. With the first generation of TKI, imatinib, one study showed that clonal evolution had no significant effect on achieving major or complete cytogenetic response, but it was a poor prognostic factor for survival in both CP and AP [24]. Another study showed that clonal evolution was associated with lower cytgenetic response rate in patients in AP with other signs of acceleration [25]. For patients on second generation of TKI therapy, cases with clonal evolution but without other features of AP had similar hematologic/cytogenetic response and overall survival to cases in CP without clonal evolution. However, when other features of AP were present, the presence of clonal evolution adversely affected overall survival [26].

As mentioned above, the prognostic significance of clonal evolution also depends on the specific type of chromosomal changes. Fabarius et al. reported that the major route, not minor route cytogenetic aberrations at initial diagnosis, had a negative prognostic impact on CML patients [27]. Consistently, a recent study indicated that trisomy 8, chromosome 17 abnormalities, and complex cytogenetic changes carried worse prognosis than others [26]. Accordingly, in European LeukemiaNet recommendations, the major routes, not the minor routes of chromosomal abnormalities, are defined as one of criteria for AP [28]. Although 11q23 abnormalities are considered as “minor route” abnormalities, they appear to carry a poor prognosis. In our cohort, nine patients (9/11) died at the last follow-up. The majority of cases had 11q23 abnormalities in BP(8/11). Only one patient (case 1) had 11q23 in CP, in the absence of other AP features. This patient achieved complete cytogenetic response and major molecular response after nilotinib treatment. Two patients (cases 2 and 3) had 11q23 in AP, accompanied with other AP features including increased blast count and thrombocytopenia. These two cases showed poor response to TKI therapy and transformed to blast phase after a period of 1.6 and 12 months.

Interestingly, not all cases with 11q23 rearrangements showed *MLL* involvement by FISH analysis. In our series, 30% (3/10) cases showed negative *MLL* rearrangement by FISH study using a commercial *MLL* breakapart probe. Although not frequent, similar findings had been described in CML and other myeloid neoplasms. Giurliano et al. reported two cases of myeloid neoplasms (AML and atypical CML) with cytogenetic changes involving 11q23 but with intact *MLL* gene by FISH: one with t(3;11)(q21;q23) and another with t(6;11)(q15;q23) [29]. Cox et al. studied 378 adult acute leukemia cases and found that in 18 cases with rearrangements involving 11q22-25 bands by conventional karyotyping, only 11 had *MLL* gene involvements [30]. In CML, two cases with 11q23 rearrangements but intact *MLL* were previously reported. One case had t(2;11)(p21;q23) [12] and the other had t(4;11)(q21;q23) [5]. In our study, three patients showed 11q23 translocations by conventional cytogenetics but were negative for *MLL* involvement by FISH, including t(2;11)(q32;q23) (case 4), t(4;11)(q21;q23) (case 1), and t(6;11)(p11.2;q23) (case 2). The exact mechanisms and roles of 11q23+/*MLL*− in leukemia are unknown. We proposed two potential mechanisms here. First, although *MLL* gene appeared intact in these cases, *MLL* gene may be controlled and dysregulated by other promoters or enhancers as a result of translocations. Secondly, other genes at the 11q23 locus may play roles in leukemogenesis, such as *FLI1* [31,32]. Future molecular study to identify the breakpoints of these 11q23+/*MLL*− translocations and their fusion partner is needed.

In case 11, although the patient initially presented with chronic phase CML and responded to TKI, he later developed blast transformation with 11q23 translocation. FISH for *BCR*-*ABL1* was negative. The development of acute leukemia in Ph-negative clones is intriguing. The occurrence of chromosomal changes in Ph-negative cells is less common than in Ph-positive cells, and the significance of this phenomenon is not fully understood [33]. Jabbour et al. analyzed 258 CML patients in CP with imatinib therapy [34]. After a median follow-up of 37 months, 9% patients developed chromosomal abnormalities in Ph-negative cells, with − Y and trisomy 8 being the most common. Others included del (7), del (20), t(11;17), etc. Although similar cytogenetic changes are associated with myelodysplastic syndromes, these cytogenetic abnormalities in CML seem transient and most disappeared after a median of 5 months. Development of MDS or acute leukemia is extremely rare in Ph-negative cells. In a study of 1,701 CML patients, Kovitz found that 3 patients developed AML and MDS in Ph-negative cells [35]. Monosomy 7 and complex cytogenetic changes were the most common chromosomal abnormalities in reported cases of MDS/AML developed in Ph-negative cells [36-40]. In our case, the patient developed AML in Ph-negative cells carrying 11q23/*MLL* abnormality. To our best knowledge, this is the first case of acute leukemia developing in Ph-negative cells with 11q23/*MLL* involvement. The mechanisms behind the development of chromosomal abnormalities in Ph-negative cells are not clear, but it is less likely due to TKI therapy [34]. A plausible explanation is that t(9;22) is not a random event, but rather develops from a preceding genetic abnormality, which occurs at the level of hematopoietic stem cells. This genetic abnormality not only induces t(9;22) but also other chromosomal abnormalities. During CML treatment, cells with t(9;22) are eradicated and cells with other chromosomal abnormalities emerge [41].

Two patients (cases 5 and 6) presented with acute leukemia with t(9;22) and 11q23 rearrangements at time of initial diagnosis. This timing raised the question of whether they were *de novo* AML or BP of CML. Features that favor the latter include the presence of i(17)(q10) and “dwarf” megakaryocytes; both are more frequently seen in CML cases. Also in case 5, the patient had basophilia and eosinophilia, a phenomenon more often encountered in CML.

As mentioned above, occasional case reports of CML with 11q23 translocations are available in the literature. The fusion partners previously reported for 11q23 in CML include 1q21 [10], 2p21 [12], 4q21 [5], 6q25 [7], 9p22 [11,8,6], 17q21 [9], 19p13.1 [14], and 19p13.3 [13]. In our study, 9p21-22/AF9 was the most common partner for 11q23. In addition, we identified several new fusion partners for 11q23 in CML: 2q32, 6p11.2, 17q25, and 18q11.2. Of these, t(11;17)(q23;q25) potentially involves the SEPT9 gene on 17q25 and had been reported in AML and myelodysplastic syndrome with a poor prognosis [42], whereas t(2;11)(q32;q23), t(6;11)(p11.2;q23), and t(11;18)(q23;q11.2) translocations have never been previously described in the literature. It will be of interest to explore and characterize the potential genes involved in these reciprocal translocations and their roles in leukemia.

In conclusion, 11q23 translocations are rare events in CML, frequently occurring in blast phase and associated with a poor prognosis. Not all 11q23 translocations involve *MLL* gene. We described a first case of acute leukemia developed in Ph-negative cells with 11q23/*MLL* involvement. Several novel genetic loci are identified as fusion partners for 11q23.

## Materials and methods

### Case selection, histologic and clinical review

We searched the pathology archives of our institution from 1998 to the present for cases of CML with available karyotypes. Conventional cytogenetics and FISH results were reviewed to identify cases with 11q23 translocations. Other genetic changes involving 11q23, such as del(11)q23, were not included in this study. Biopsy and smear slides were retrospectively reviewed. The corresponding medical records were reviewed to obtain clinical data, including age, sex, disease progression, treatment regimens, response to therapy, and follow-up data.

### Cytogenetic studies

Conventional chromosomal analysis was performed on G-banded metaphase cells prepared from unstimulated 24- and 48-h BM aspirate culture using standard methods. Twenty metaphases were analyzed and the results were reported using the International System for Human Cytogenetic Nomenclature (ISCN 2013). FISH for *MLL* gene rearrangement was conducted on BM culture cells or direct aspirate smears using *MLL* dual color, breakapart probe (Abbott Molecular, Inc.) according to the standard protocols.

### Definition of cytogenetic and molecular response

Complete cytogenetic response is defined as 0% Ph-positive metaphases using conventional cytogenetics analysis of at least 20 metaphases. Major molecular response is defined as more than 3-log reduction of *BCR*-*ABL* mRNA.

### *BCR*-*ABL1* transcript measurement

*BCR*-*ABL1* and *ABL1* transcript levels were detected simultaneously and quantitative results expressed as the ratio of *BCR*-*ABL1* to *ABL* levels.
